# Re‐make, re‐model: evolution and development of vertebrate cranial lateral lines

**DOI:** 10.1111/brv.70045

**Published:** 2025-06-07

**Authors:** Vishruth Venkataraman, Marco Lopez, Victoria E. Prince, Michael I. Coates

**Affiliations:** ^1^ Department of Organismal Biology and Anatomy University of Chicago Chicago Illinois 60637 USA

**Keywords:** lateral line, placode, neuromast, dermoskeleton, vertebrate, gnathostome, phylogeny

## Abstract

Lateral lines are placodally derived mechanosensory systems on the heads and trunks of many aquatic vertebrates. There is evidence of lateral lines in the earliest known vertebrate fossils, and they exist in organisms with widely different craniofacial morphologies – including the presence or absence of jaws, external or internal nostrils, and variable positions of the cranial cartilages with respect to eyes and braincase. Consequently, the lateral lines make an ideal study system to understand how morphological variation in a deeply conserved sensory system responds to overall evolution of the head. However, palaeontological and developmental data have not been integrated to elucidate the history of this system in the context of evolving vertebrate crania. The emergence of new imaging techniques and molecular methods to study ontogeny in non‐model systems provides unique opportunities for such a study. This review examines open questions in light of new fossil discoveries that have altered our understanding of vertebrate evolution as well as new insights on the development of non‐model taxa. We find that the diversity of lateral lines is not the result of simplification from a complex ancestral condition as previously supposed. Rather, the anterior lateral line systems of living gnathostomes result from an evolutionary episode of reduction and reassembly, both preceding and overlapping the origin of jawed vertebrates. This event is coupled to a marked postorbital to orbital–preorbital shift in the territorial elaboration of the lateral line systems, and we argue that this spatial move likely signals functional change, coinciding with a major enhancement of the gnathostome vestibular system.

## I. INTRODUCTION

**Fig. 1:**
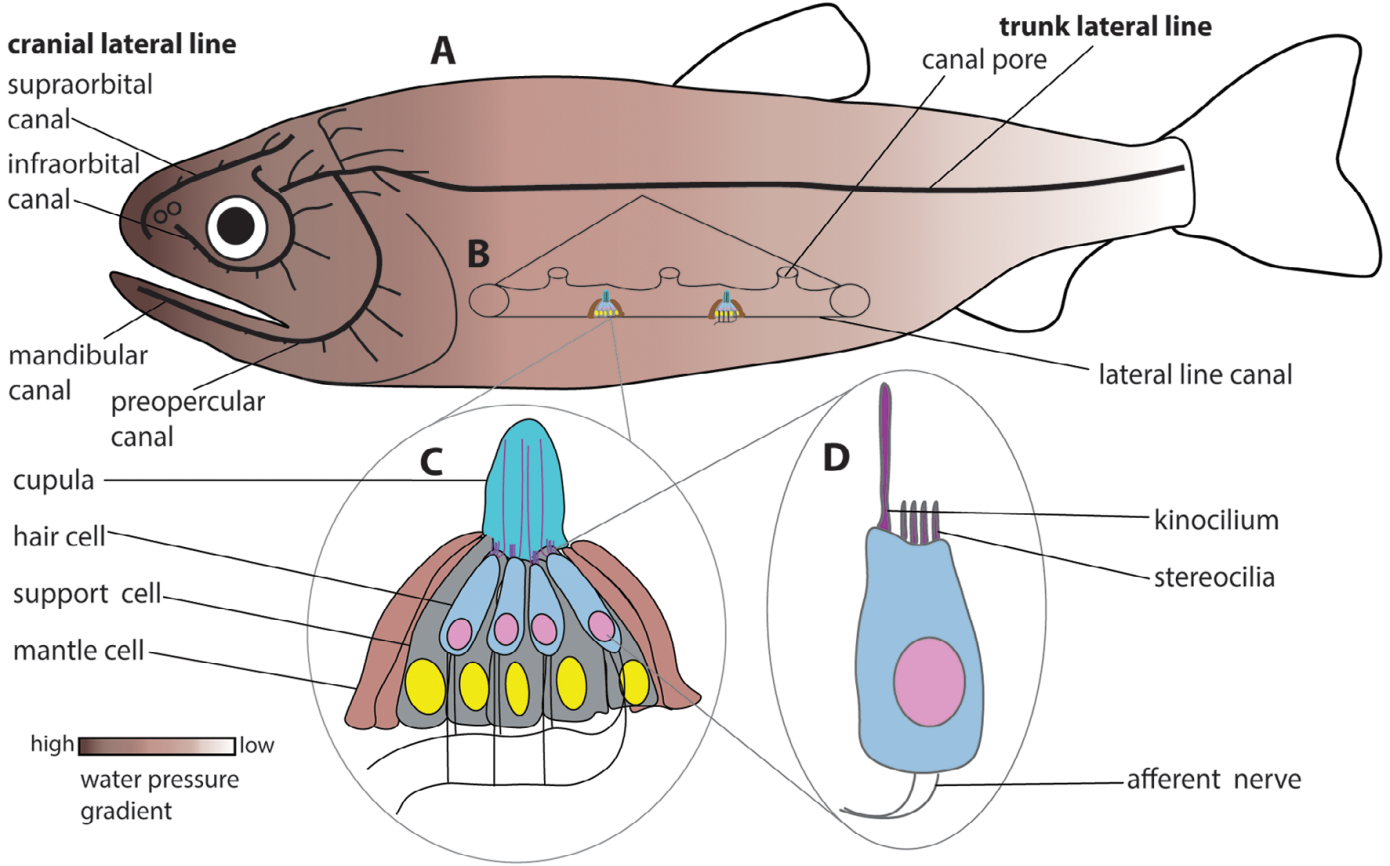
Basic structure of lateral lines. (A) Model of a teleost fish adapted from Hilton (2002) showing components of cranial and trunk lateral line networks (black). Colour gradient indicates distribution of water pressure across body, after Mogdans (2019). (B) Detail of trunk canal section illustrating neuromasts between pores opening externally. (C) Neuromast structure: hair cells surrounded by support cells, enveloped by layer of mantle cells; hair cell sensory cilia extend into jelly‐filled cupula. (D) Single hair cell showing kinocilium and stereocilia, connected to afferent nerve.

Both cranial and trunk lateral lines develop from a suite of cranial neurogenic placodes. These lines are present in all lineages of aquatic vertebrates with the exception of amniotes. This system is composed of specialized receptive organs called neuromasts, which are contained within canals or grooves or embedded superficially in the skin, bones, and scales of the head and trunk (Fig. 1A). These neuromasts consist of specialized hair cells surrounded in some cases by support cells (Fig. 1B, C). The hair cells have a prominent kinocilium and a series of smaller stereocilia projecting into a jelly‐filled cupula (Fig. 1D). These neuromasts are exposed to water in a lateral line canal, or in a groove, or directly from the surface of the skin in shallow pits. Different neuromasts on different parts of an organism's body contain hair cells polarized in characteristic orientations. Water currents deflect the cilia differentially and this deflection is then transduced as nervous impulses, transmitted from the neuromast organ to the hindbrain *via* afferent nerves.

Fishes use lateral line systems to perceive hydrodynamic stimuli at macro‐ and microscales (Mogdans, 2019). These systems have traditionally been implicated in schooling and orientation to water currents: fish that have had their lateral line network ablated are unable to orient themselves to the direction of bulk water movement (Liao, 2006). Species‐specific adaptations of the lateral line can also filter out mechanosensory stimulation by strong currents, allowing the fish to detect high‐frequency stimuli like approaching predators (Herzog, Klein & Ziegler, 2017). However, recent functional studies have also shown that this mechanosensory system is part of a broader multimodal “bioacoustic” network working in concert with the ear to detect auditory signals in the water (Higgs & Radford, 2013).

The study of lateral lines has attracted the interest of three distinct research programmes: palaeontologists and comparative anatomists concerned primarily with the *pattern* and disparity of lateral lines across vertebrate lineages (Northcutt, 1989), functional biologists and neuroscientists researching how lateral line input is processed and behaviours modified in response (e.g. Liao, 2006), and developmental biologists concerned with the *process* generating lateral line morphologies in a few easily accessible model taxa (Dambly‐Chaudière, Cubedo & Ghysen, 2007). Developmental studies have primarily been concerned with the posterior or trunk lateral line, that is the component posterior to the otic vesicle. By contrast, palaeontological and functional studies have primarily been focused on the anterior or cranial lateral lines that occupy the skull and head shield. Importantly, data from all three programmes are reciprocally informative. Given our rapidly changing picture of early vertebrate evolution – thanks to new fossil discoveries, new imaging tools and new methods of phylogenetic reconstruction – developmental data gleaned from extant taxa can be used to improve our understanding of lineage‐specific patterns of developmental bias or phylogenetic constraint. Reciprocally, comparative embryological approaches in a phylogenetic framework can reveal the developmental mechanisms underlying varied lateral line morphologies. This review focuses on these two approaches: evolution and development of cranial lateral lines.

Palaeontologists have a long‐standing interest in lateral lines because of the high likelihood of their preservation within the dermal skeletons of early vertebrates. Furthermore, lateral lines exhibit considerable morphological variation across different lineages. Thus, lateral lines provide a data‐rich window on sensory system evolution within the context of major morphological transitions. Examples of these transitions include the advent of jaws and changes in jaw position, changes in the number and location of nostrils, and changes in cranial proportion and position of the eyes. Further, vertebrates have also undergone major ecological transformations: from benthic to nektonic to marginal habitats and from demersal to pelagic and emergent lifestyles. Finally, lateral lines have been linked to hypotheses of dermal bone homology (Moy‐Thomas, 1941; Parrington, 1949; Rizzato *et al*., 2020; Schultze, 1993; Thompson, 1993; Westoll, 1941) and thus feed directly into analyses of phylogenetic relationships.

In parallel, lateral lines are of great interest to developmental biologists. This is largely because the posterior or “trunk” lateral line cells of zebrafish (*Danio rerio*) provide a tractable model for the study of collective cell migration (Haas & Gilmour 2006; Chitnis, Dalle Nogare & Matsuda, 2012; Dalle Nogare *et al*., 2017). The molecular mechanisms underlying the migration and morphogenesis of posterior lateral line neuromast primordia have been delineated through myriad studies. However, the more complex and variable anterior (or head) lateral lines have received much less attention (Iwasaki *et al*., 2020). This is especially significant because current data indicate that the head and trunk lines, at least in zebrafish, are patterned by different developmental mechanisms. But, the zebrafish is just one of nearly 35,000 species of extant teleost fishes (Froese & Pauly, 2024) separated from other bony fishes and cartilaginous fishes by nearly half a billion years of evolution (Zhu *et al*., 2022). Given this evident dearth of comparative embryological data, the general *versus* derived modes of lateral line patterning and development across the vertebrates present an area ripe for exploration.

The goals of this review are to re‐examine the evolution of the lateral line system in light of new phylogenies and data, both morphological and developmental. We include a discussion of how major evolutionary changes such as the occupation of the water column, the advent of predation, and transformations of cranial anatomy are linked to the origin and disparity of lateral line systems in living taxa. Further, we highlight gaps in the comparative data – taxonomic, morphological and developmental – with a view to setting an agenda for future research. Finally, we identify avenues of study in lateral line evolution that could be answered *via* an integrative approach combining comparative embryology, functional studies, digital visualization, and new phylogenetic analyses. Key terms used in this review are defined in Table 1.

**Table 1:** Glossary of key terms.

Crown group	A monophyletic group defined by living representatives of a lineage, their most recent common ancestor and all extinct descendants.
Macromery	The condition, for example seen in osteichthyans, of the dermal skeleton being composed of conserved numbers of large plates
Micromery	The condition, for example seen in sharks, of the dermal skeleton being composed of many small units (such as placoid scales).
Monophyletic group	A natural group containing the most recent common ancestor and all descendants of that common ancestor.
Paraphyletic group	A group descended from a common ancestor but with incomplete membership.
Placode	A thickening of the embryological epithelial ectoderm that gives rise to various sensory organ systems like the nose, ear or lateral line.
Plesiomorphy	A trait or characteristic defining a group at a higher rank (i.e. more inclusive) than that of the group under consideration.
Polytomy	An unresolved relationship in a phylogenetic tree represented by three or more branches extending from a single node.
Stem group	A paraphyletic assemblage of extinct taxa most closely related to, but not included within, a crown group.
Synapomorphy	A uniquely shared derived trait or characteristic uniting a group with another to which it is most closely related.
Total group	An assemblage composed of the crown group plus stem group.

## II. VERTEBRATE LATERAL LINE DIVERSITY

### (1) General conditions

**Fig. 2:**
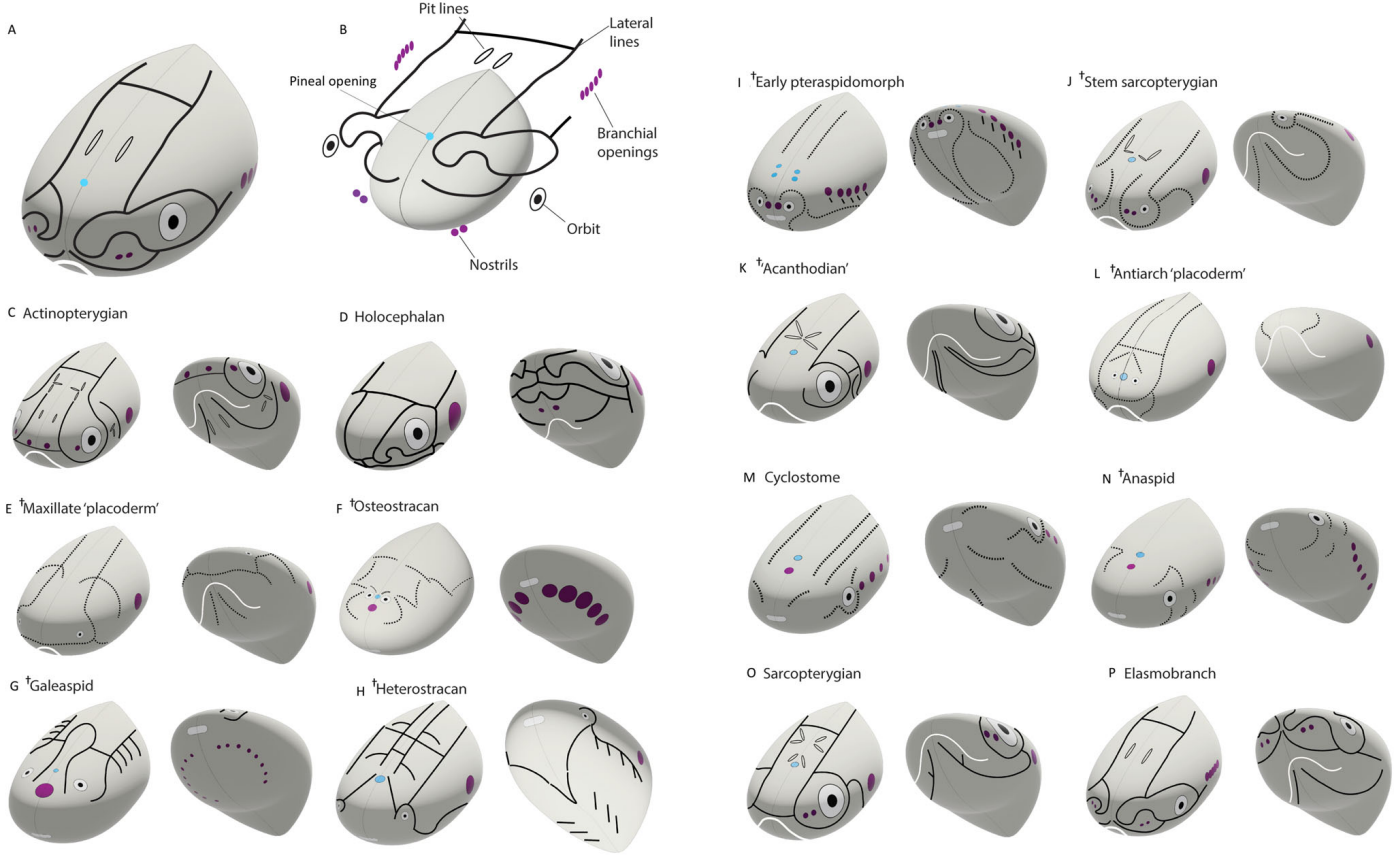
Extant and extinct vertebrate cranial models. Each is abstracted from multiple representatives of the taxon in question to illustrate features of the characteristic lateral line networks, highlighting system diversity. A and B provide key to panels C–P. Taxon panel order follows the sequence of taxa discussed in the main text. (A) Elasmobranch head in dorsolateral view. (B) Same head “exploded” to show component parts. (C) Actinopterygian, after Gardiner (1984), with teleost pit‐line pattern after Lekander (1949). (D) Holocephalan after Cole (1897). (E) Maxillate “placoderm” after Zhu *et al*. (2016). (F) Osteostracan after Janvier (1974, 1985). (G) Galeaspid after Shan *et al*. (2020). (H) Heterostracan (cythaspid and pteraspid conditions) after Janvier (1996) and Randle *et al*. (2022). (I) Early pteraspidomorph (†Astraspid, †Arandaspid and †Sacabambaspid conditions) after Gagnier *et al*. (1986), Sansom *et al*. (1997) and Pradel *et al*. (2006). (J) Stem sarcopterygian (†*Guiyu* and †*Psarolepis* conditions) after Yu (1998), Zhu *et al*. (1999) and Qiao & Zhu (2010). (K) “Acanthodian” generalized after Watson (1937). (L) Antiarch “placoderm” after Graham‐Smith & Parrington (1978). (M) Cyclostome (*Lampetra*) after Marinelli & Strenger, 1954. (N) Anaspid (stem‐cyclostome) after Smith (1957). (O) Crown sarcopterygian *Gogonasus* (stem‐tetrapod) after Long *et al*. (1997). (P) Crown elasmobranch (squalomorph), after Garman (1888) and Johnson (1917). Use of a dagger indicates an extinct taxon.

The disparate patterns of lateral line networks in major extant and extinct vertebrate groups are summarized in Fig. 2. This figure is a synthesis of several representative published descriptions for each group and shows an inferred general condition for each. Morphological features of the various kinds of heads have been abstracted to show the relationship of lateral lines to major landmarks including the eyes, nose, and the mouth (Fig. 2A, B). The order of taxa displayed in Fig. 2 follows their relative familiarity rather than phylogenetic convention, hence, teleosts are presented first.

The general condition in extant jawed vertebrates, as seen for example in a representative teleost (Figs 1A and 2C), consists of one main trunk canal running from behind the head to the caudal fin, often referred to as the posterior lateral line. Anteriorly, this line extends as several branches coursing around various cranial openings like the orbits (Figs 1A and 2C), the nostrils and the operculum, as well as extending along the jaw. These anterior lines are consequently termed the orbital, nasal, pre‐opercular and mandibular branches of the lateral line network. This basic pattern is modified or elaborated in different lineages. For example, teleosts, in addition to having elaborated lines (Webb, 1989), can have accessory lines of superficial neuromasts flanking the main head canals (Northcutt, Holmes & Albert, 2000) (Fig. 2C), and cartilaginous fishes often have additional canals and loops as seen in the rostrum of holocephalans (Didier, 1995) (Fig. 2D) or the pectoral fins of batoids (Abe *et al*., 2012). In some cases, like amphibians or bathypelagic fishes, all lateral lines are composed of neuromasts within grooves in the skin as opposed to canals. However, to understand fully the extent and evolution of vertebrate lateral line diversity, we need to delve into the fossil record.

A selection of exemplar fossil vertebrates is included in Fig. 2 to demonstrate the historical diversity of anterior lateral line systems. †“Placoderms” are the earliest jawed vertebrates. Use of a dagger in this context denotes an extinct taxon, and the use of inverted commas denotes uncertainty about whether “placoderms” constitute a natural group or merely a grade of extinct vertebrates. Nevertheless, †“placoderms” are mostly characterized by the possession of cranial and thoracic skeletons consisting of large plates which preserve clear traces of their lateral line systems. These fishes had diverse morphologies, ranging from large, predatory open‐water swimmers like the arthrodires (for example, †*Dunkleosteus*) to small, benthic taxa including †antiarchs such as †*Bothriolepis* (Janvier, 1996). Recently discovered maxillate “placoderms” (Fig. 2E), including the Silurian †*Entelognathus*, are inferred to be “†placoderms” but are also considered to be the sister group of extant jawed vertebrates. Significantly, these are the only “placoderms” known to have lateral lines on the lower jaw (Zhu *et al*., 2016). It follows that †*Entelognathus* occupies a key position in the transition from stem to crown jawed vertebrates, and the maxillate “placoderms” have prompted a significant reappraisal of relationships among taxa close to the crown gnathostome node (Zhu *et al*., 2016, 2013; King *et al*., 2017; Li *et al*., 2021).

Importantly, beyond “placoderms”, the evolutionarily deeper stretches of the gnathostome lineage also include a host of jawless groups. Many of these jawless fishes also bear a head shield consisting of large plates and a tail covered in bony scales, thereby extending the total evolutionary record of lateral line system diversity. †Osteostracans (Fig. 2F) and †galeaspids (Fig. 2G) have dorsoventrally flattened crania, enclosing remarkably detailed internal morphology (Janvier, 1985; Gai *et al*., 2011). By contrast, †heterostracans, including †pteraspids (Fig. 2H), have head shields but no substantial endocranial remains (Moy‐Thomas & Miles, 1971). This deficit in the quality of fossil material extends to the very earliest vertebrate body fossils, including †*Astraspis*, † *Arandaspis* and †*Sacabambaspis* (Fig. 2I), all from the Ordovician period (Ritchie & Gilbert‐Tomlinson, 1977; Sansom *et al*., 1997; Gagnier, Blieck & Rodrigo, 1986; Pradel *et al*., 2006; Dearden *et al*., 2023).

From these data, we infer that the general condition for vertebrates is to have one or a series of longitudinal (or trunk) lines extending from approximately the otic region to the caudal end of the animal. Rostrally, extensions of these lines course around apertures in the skull, including the orbits, the nostrils, the mouth and gill openings. However, the eyes of the earliest vertebrates were positioned close to the front of the head (Gagnier *et al*., 1986). Thus, the lateral line system in these fossils consists of multiple longitudinal lines behind the orbits, often with multiple transverse extensions. These postorbital and dorsal networks are elaborated in †heterostracans and †galeaspids (Fig. 2G, H), where such lines may form complex reticulating networks (Janvier, 1996; Denison, 1964; Yang *et al*., 2024).

### (2) Orbital lines

Orbital lines in modern jawed vertebrates generally consist of a supraorbital and infraorbital line coursing around the eye. Although the two lines are connected caudally in most extant jawed vertebrates, fossil data from early sarcopterygians (Qiao & Zhu, 2010) (Fig. 2J), actinopterygians (Gardiner, 1984) (Fig. 2C) and “†acanthodians” (Fig. 2K) (early sharks) (Burrow, 2021; Watson 1937) demonstrate that the primitive condition is the two lines being disjunct caudally. In these cases, the supraorbital lines lie parallel to a stretch of an infraorbital line behind the eye (Fig. 2C). Therefore, in modern taxa, the connections between two orbital lines both behind and in front of the eyes represent a derived condition but it remains unclear how many times these connections evolved.

Orbits have been modified in both position and size during vertebrate evolution, and the supraorbital and infraorbital lines have shifted accordingly with respect to orbit location as well as the position of a pineal opening. We note that infraorbital lines are the most consistently present component of the anterior lateral line system throughout vertebrate diversity. The earliest vertebrate condition, as revealed by †heterostracans, †galeaspids and †antiarch “placoderms” is to have supraorbital lines converging behind the pineal opening (Fig. 2H, G, L). In †osteostracans, the pineal opening is located anteriorly, between the eyes, consequently reducing the space for a post pineal commissure, and correlates with a total absence of supraorbital lines (Fig. 2F). Similar instances occur in lampreys (Fig. 2M), where the pineal is also anterior to the eyes. However, the †anaspids (stem group cyclostomes) are poorly preserved showing few traces of a rudimentary line network (Fig. 2N). In living gnathostomes (osteichthyans and chondrichthyans: e.g. Figure 2O, P), orbits are in a much more lateral position relative to the orbits of †osteostracans and †galeaspids and the supraorbital lines connect with the main trunk lines on their respective sides. In some cases, a medially directed supratemporal canal connects the trunk lines of both sides, forming an occipital commissure, posterior to the supraorbital canals.

Pit lines are short grooves in the skin that house superficial neuromasts. In extant forms, the pit lines are generally found in the parietal/postparietal domain. They also occur in extinct taxa, such as the lines found on the nuchal bones of “†placoderms” and the cheeks and jaws of early osteichthyans. The anterior pit line in modern osteichthyans is a short, open groove with superficial neuromasts positioned behind the caudal extremity of the supraorbital line. There are usually two other pit lines, termed the middle and posterior lines, radiating out from the crown of the head. Unlike the anterior pit line, the middle and posterior pit lines do not bear any obvious spatial relationship to other parts of the anterior lateral line system.

### (3) Cheek and jaw lines

The general condition for lines on the cheek of jawed vertebrates is a series of longitudinal and transverse lines posterior to the orbit. This includes, as seen in zebrafish, lateral lines on the preopercle as well as lines on the lower jaw: the mandibular line (Fig. 1A). Embryological evidence from several taxa including zebrafish supports morphological data that connect the lower jaw line to a preopercular line: one is a continuation of the other (Iwasaki *et al*., 2020; Lekander, 1949). The cheek region accommodates the jaw adductor muscles of gnathostomes and is therefore also a derived feature of the group (although rarely recognized as such). Lampreys and other jawless vertebrates do not have a well‐defined cheek as such, so the relationship of their postorbital lines to those of jawed vertebrates is unclear. The mandible (lower jaw) is a feature of jawed vertebrates, but mandibular lines are not present in early members of the clade, hence the significance of the mandibular lateral line discovered in †*Entelognathus* (Zhu *et al*., 2013).

### (4) Sensory system hierarchy and new morphologies

We note that there appears to be a hierarchy in sensory system patterning, with lateral lines shifting to accommodate the changing positions of other cranial sensory systems, particularly the olfactory, visual, and hearing systems. Novel and extreme morphologies help us to understand such apparent hierarchies. For example, body forms can be flattened either dorsoventrally or laterally. Dorsoventral compression may be restricted to the rostrum, as in sturgeons and paddlefish, or involve flattening of the entire body, as in the skates and rays. Lateral flattening can involve extreme asymmetry, as seen in flatfishes (pleuronectids). In each instance, the lateral line system invades new territory, although to different degrees in different lineages. In addition to skates and rays, several other chondrichthyan groups have evolved flattening, including angel sharks and wobbegongs (carpet sharks). In most cases, lateral lines have grown onto the expanded, flattened regions of the body, as seen for example in guitarfish (Garman, 1888). These extended lateral line branches, often misleadingly called scapular lines, run from the main trunk line and grow out laterally (Maruska, 2001). The most extensive flattening in skates and rays involves fusion of the pectoral girdle to the rostral tip of the snout, and in this unique instance, the cranial lateral line canals extend onto the pectoral fin (Ewart & Mitchell, 1895; Maruska, 2001). Notably, these pectoral lines are also unique in being the only example of a rostral (preotic) placode‐derived lateral line growing in a posterior direction.

**Fig. 3:**
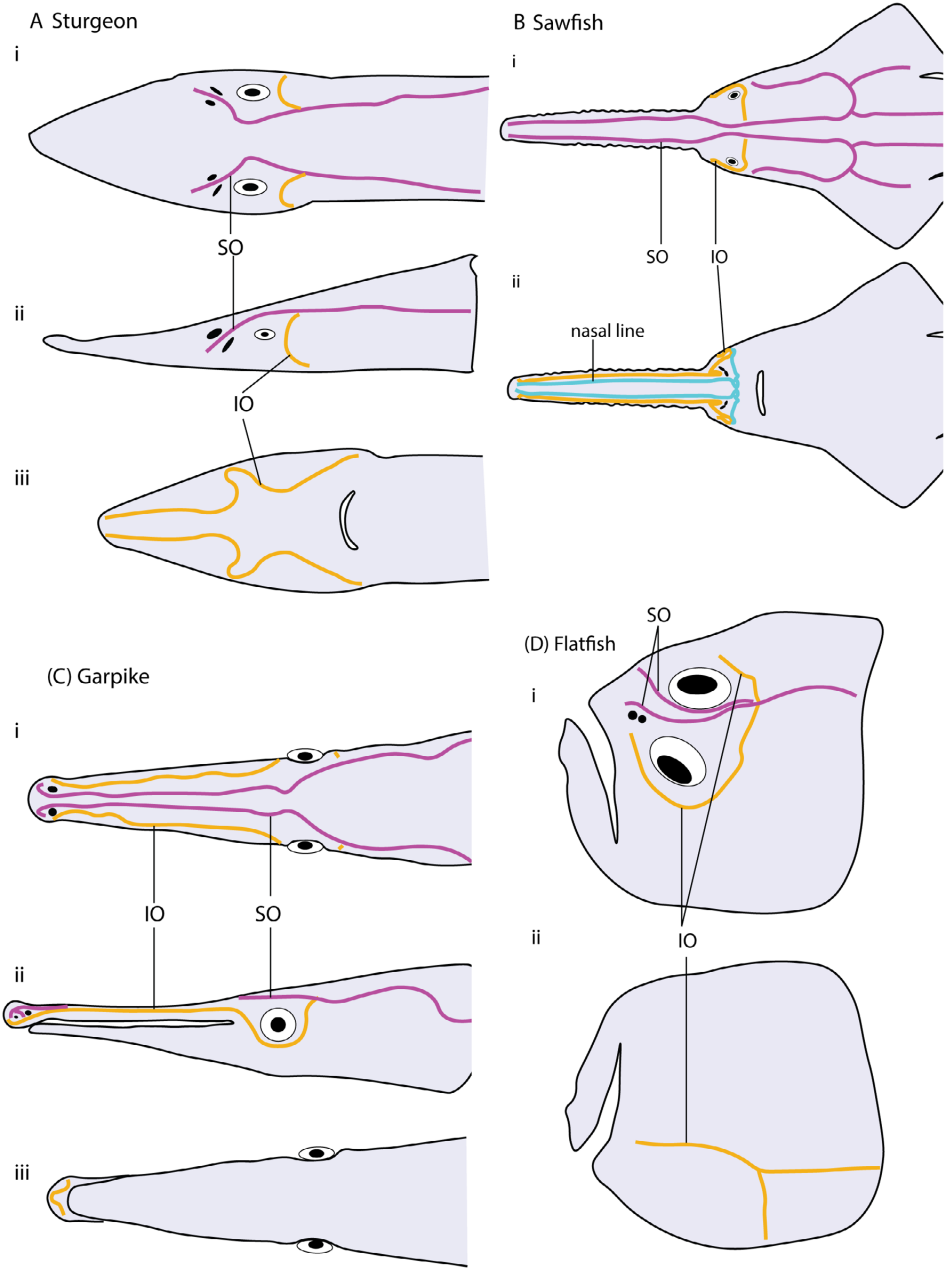
Orbital lines on extreme head shapes. (A–C) Examples of rostral elongation showing the relationship of orbital lateral line canals to nostrils. (A) Sturgeon after Gibbs & Northcutt (2004); (i) dorsal view; (ii) lateral view; (iii) ventral view. (B) Sawfish after Wueringer *et al*. (2011); (i) dorsal view; (ii) ventral view. (C) Garpike after Song & Northcutt (1991); (i) dorsal view; (ii) lateral view; (iii) ventral view. (D) Flatfish (*Tephrinectes sinensis*) after Voronina *et al*. (2019); (i) dorsal view; (ii) ventral view. Supraorbital line (magenta, SO) terminates close to eyes in sturgeons, and extends to rostral tip in sawfishes and garpikes. Infraorbital line (yellow, IO) extends to rostral tip in all cases. Sawfishes have “nasal” line characteristic of elasmobranchs (blue). Nostrils are shown in black throughout.

Elongate rostra, not always associated with elongate jaws, repeatedly evolved in both osteichthyans and chondrichthyans. In osteichthyan examples such as sturgeons and paddlefish, and chondrichthyan examples such as sawfishes and sawsharks (Wueringer *et al*., 2011), the rostrum is elongate, but the mouth is in a sub‐terminal position with the nostrils close to the face (Fig. 3A, B). However, despite these similarities in gross morphology, the distribution of the orbital lateral lines differs between these two groups. In sturgeons and paddlefish the infraorbital canal extends onto the ventral side of the snout, forming complex loops, but the supraorbital line terminates abruptly in a proximal position between the nostrils (Fig. 3A). By contrast, in sawfishes and sawsharks, both lines run to the anterior tip of the snout (Fig. 3B). It appears that nostril position dictates these different patterns of lateral line distribution. In sturgeons and paddlefish, the supraorbital line lies between the anterior and posterior nostril and is perhaps trapped in this proximal domain. In sawfishes and sawsharks, however, both nostrils lie outside the loop of the orbital lines, and therefore the lines are not restricted by nostril position. Conditions in garpike (Fig. 3C), another osteichthyan, corroborate this restriction scenario. Garpike have elongated rostra, but these fish have both nostrils positioned at the very tip of the snout. Here, both supra and infraorbital lines extend all the way to the distal extremity and loop around the nostrils. Taken together, these three conditions across two divisions of jawed vertebrates support the idea that lateral lines have their positions dictated by cranial openings such as nostrils. In this regard, the migrating orbits of pleuronectids might provide a further test of the generality of this scenario, in which lateral line network patterning is warped to match the location of other major sensory systems.

Flatfishes (pleuronectids, Fig. 3D) develop as bilaterally symmetrical larvae, but through ontogeny begin to lie on one side (the “blind side”) and move their orbits, nostrils and mouth towards the side facing the water column (the “eyed side”) (Voronina, Sideleva & Hughes, 2019). During this process, a conventionally symmetric larval lateral line system becomes asymmetric. Recent data (Duarte‐Ribeiro *et al*., 2024) indicate that cranial asymmetry evolved once in flatfishes, and extensive studies (Voronina *et al*., 2019; Harvey, Blaxter & Hoyt, 1992; Neave 1986) have demonstrated that all members of the group show varying degrees of asymmetry in lateral line positioning. While the morphological diversity of pleuronectiform lateral lines has been documented in detail, there is scant information on how developmental remodelling emerges within the system. It is unclear if neuromasts undergo apoptosis, with new ones arising within the transformed head, or if canals and neuromasts somehow relocate along with a shift of the dermis or change in cranial morphology.

Yet another example of the hierarchical relationship between lateral lines and other sensory systems is evident in the internalization of the posterior nostril in tetrapods, lungfishes, and holocephalans (also known by a variety of other names including chimaeras, ghost sharks, ratfish and elephant sharks). The ancestral condition for lobe‐finned osteichthyan lineages, as exemplified by the early tetrapod †*Tungsenia* (Lu *et al*., 2012) and early lungfish †*Porolepis* (Kulczycki, 1960; Chang, 1995), was for the orbital line to loop around and encompass both nostrils. However, in intermediate forms like the early tetrapod *Kenichthys*, where the posterior nostril is at the ventral border of the maxilla, the supraorbital line displays a kink, as if it were dragged down with the nostril (Zhu & Ahlberg, 2004). In the case of extant lungfishes both nostrils are internal, with the anterior nostril on the medial border of the lip. Here, both nostrils appear to have “broken through” the loop of the supraorbital line, which never connects with the infraorbital line. In holocephalans the posterior nostril is once again within the medial border of the upper lip (Howard *et al*., 2013) but in this instance both nostrils lie outside the loop of the orbital lines, thus no lines are altered or interrupted.

In summary, lateral lines appear to be subordinate or secondary to other sensory systems. The positioning of cranial openings for these systems, like the olfactory or optic, influences the course of the anterior lateral lines. It follows that the phylogenetic legacies of chondrichthyans and osteichthyans – the initial positions of their nostrils relative to the lateral lines – played a major role in determining lateral line morphology.

## III. LATERAL LINES AND SURROUNDING TISSUES

Lateral lines derive from ectodermal thickenings or placodes in front of and behind the otic vesicle. The preotic placodes form primordia that migrate or elongate anteriorly to form the anterior lateral line network. By contrast, the placodes posterior to the otic vesicle form primordia that elongate or migrate caudally to form the posterior or trunk lateral line system.

A long‐standing area of research has focused on the relationship between lateral line systems and the surrounding dermoskeleton; for a recent review, see Hamm & Gross (2025). Westoll (1936, 1937) argued that the bones carrying lateral line canals were less morphologically variable than other dermal bones; he termed these non‐canal bones “anamestics” or filler bones (Hilton, Grande & Bemis, 2011). This observation led to a suggestion that the lateral line system developmentally induces ossification of the surrounding dermal bones (Lekander, 1949; Holmgren & Pehrson, 1949). However, Moy‐Thomas (1941) ablated the rudiments of the lateral line on one side of the head of an early larval rainbow trout (*Onchorynchus myskiss)* and reported the complete development of frontal bones on both the ablated and unablated control sides, implying that lateral lines are not necessary for dermal ossification to occur. However, neuromasts are known to regenerate after ablation (Williams & Holder, 2000). It is thus possible that neuromasts on the ablated side in Moy Thomas' (1941) experiments regenerated to allow dermal ossification to occur normally.

Lateral lines might yet be found to influence morphogenesis of specific components of the cranial dermal skeleton. Studies dating back to Allis (1889) indicate that in many fishes the dermal skull bones are produced by two separate developmental modules: a flat, membrane‐bone component and a separate cylindrical component surrounding the neuromasts (see Fig. 11 in Grande & Bemis, 1998). These two ossifications may fuse through ontogeny, forming a single dermal bone (Tarby & Webb, 2003; Webb & Shirey, 2003). The cylindrical component forms as the epithelium underlying the neuromasts invaginates to form a gully, which may then enclose the neuromasts as the apices of the gully contact each other to form a closed tube or canal. Neuromasts might have some inductive potential on the ossification of these grooves and canals. Chang & Franz‐Odendall (2014) laser‐ablated developing infraorbital neuromasts of larval and juvenile zebrafish, then analysed ossification of the infraorbital bones after the neuromasts had regenerated. They found that canal wall ossification was significantly reduced in all cases, despite the fact that the neuromasts had regenerated, although the overall shape of the infraorbital bones remained unaffected. Based on these findings, the authors inferred that neuromasts have an early inductive effect on the later ossification of the canal wall, but development of the remaining dermal bone is independent of neuromast influence.

While many studies have examined the histological structure of fossil vertebrate dermal skeletons (Keating & Donoghue 2016; Giles, Rücklin & Donoghue, 2013; Donoghue, 2002; Sansom *et al*., 1992), it is notable that there have been few studies on the histology of fossil lateral line canals or grooves. Further work is needed to explore the evolution of lateral line histology, including the taxonomic extent and history of separate lateral line canal ossifications.

In many chondrichthyans, which lack large dermal plates and have a dermal skeleton made of small scales, those scales abutting the lateral line canals are modified. In holocephalans, the only scales that remain on the trunk resemble incomplete curtain rings, forming a flexible gutter housing the lateral line (Cole, 1897; von Lubitz, 1981; Didier, Kemper & Ebert, 2012). In many †“acanthodians” (early sharks), scales surrounding the lateral lines are larger than other scales of the flank and fins (Watson, 1937) and may be morphologically distinct (Burrow *et al*., 2018; Hanke & Wilson, 2006). All these data suggest that the lateral line might influence the development and morphogenesis of adjacent scales. We suggest this observation deserves further experimental evaluation.

Current comparative data are equivocal on whether canals or grooves were the ancestral condition for vertebrates: available fossil data are insufficient. However, for crown jawed vertebrates canals appear to be the ancestral condition. But, once again, we do not know if canals evolved at the root of all vertebrates or among the earliest members of the gnathostome lineage. Resolution of this question is hindered by ongoing uncertainty concerning the monophyletic *versus* paraphyletic status of “placoderms”. Furthermore, it remains unclear if a separate bony lining around the lateral line canals is an osteichthyan synapomorphy. Finally, experimental data (Chang & Franz‐Odendaal, 2014), considered together with the observations on scale patterns in chondrichthyans, suggest a possible inductive relationship between lateral lines and mineralized portions of the dermoskeleton.

Embryological studies have explored the interaction between the lateral line and adjacent tissues early in development, particularly to test if the migratory substrate plays a role in guidance or patterning of lateral line systems. Smith, Lannoo & Armstrong (1990) attempted to delineate a potential substrate‐driven lateral line guidance mechanism in axolotl (*Ambystoma mexicanum*) embryos. They surgically manipulated the overlying epidermis in front of a migrating posterior primordium, turning it by 90 or 180°. When the epidermis was flipped by 180°, the primordium migrated indistinguishably from controls that had undergone sham surgeries, suggesting the rostrocaudal directionality of overlying tissues was unimportant. However, when the epidermis was turned by 90°, the primordium ceased to migrate, or turned at the site of surgery and wandered until it encountered the path of another primordium, whereupon it followed the new path. This suggests that migrating primordia follow “tracks” in the overlying substrate. A complete rostrocaudal flip of these supposed tracks does not change primordium migration, suggesting that the tracks are not polarized. While the molecular nature of these tracks and their interactions with the primordium remain unclear, extirpation of the underlying mesoderm (one somite) stalls primordium migration, even with no surgical flipping of the ectoderm. This suggests that the migration is influenced by both overlying epidermis and underlying mesoderm. We speculate that the track in the axolotl mesoderm might be of a chemokine molecule, as has been shown in zebrafish (Haas & Gilmour, 2006; see below for details). A crucial limitation of these studies is that they have not been replicated for the head. Thus, while these classical embryological approaches have provided important insights into how the trunk lateral line system develops, we have a much more rudimentary understanding of anterior lateral line development.

**Fig. 4:**
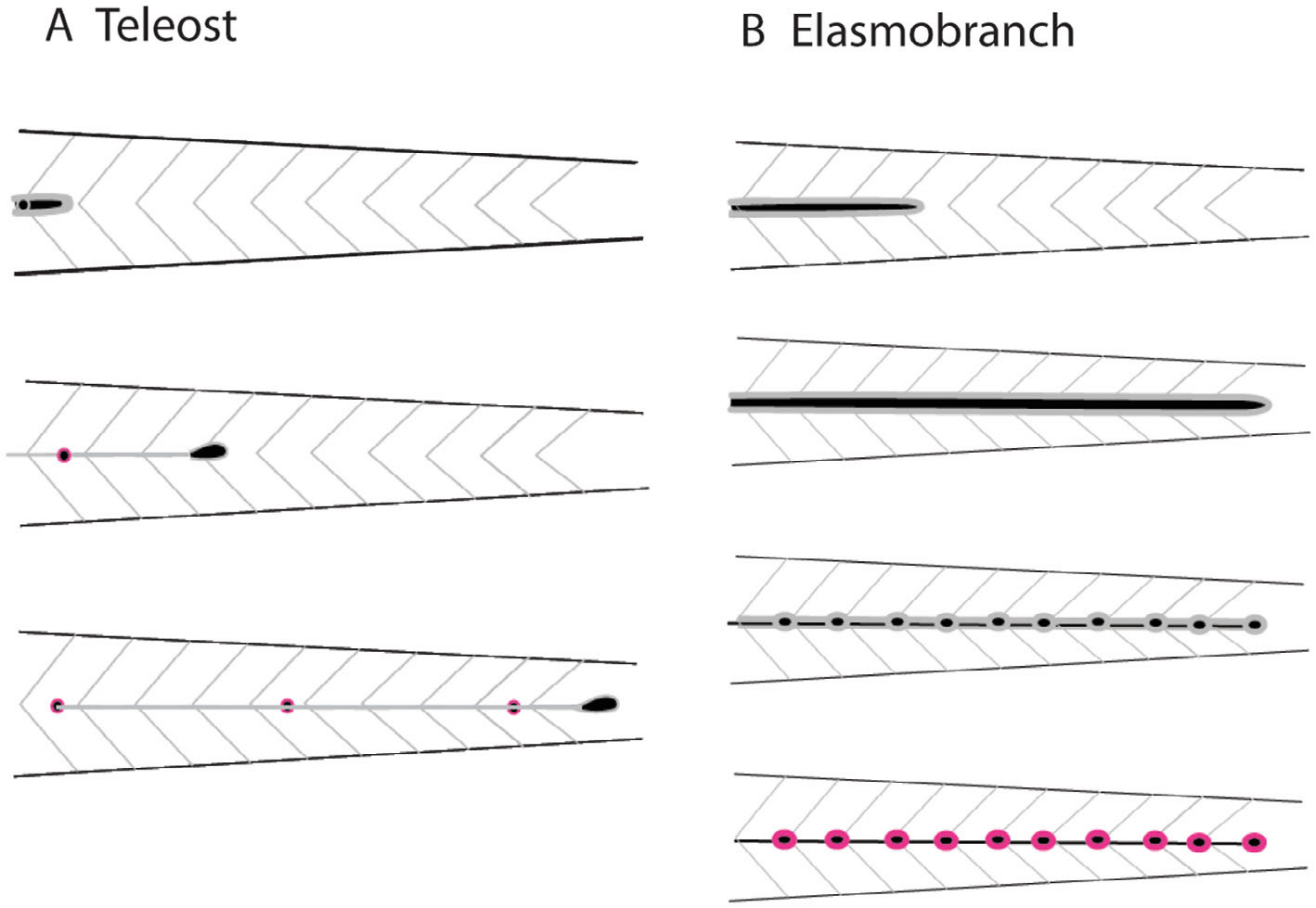
Migrating and elongating modes of lateral line development. (A) The trunk line of the zebrafish, a well‐studied teleost, develops from a migrating primordium that follows a chemokine track along the horizontal septum of the trunk paraxial mesoderm. The primordium proliferates as it migrates, forming a rosette of cells (red) at the trailing edge. Rosettes are deposited sequentially in the wake of the primordium to form neuromasts. By the time the primordium reaches the caudal end of the animal, the entire primary set of neuromasts has been deposited at appropriate locations along the trunk. (B) Most chondrichthyan lateral lines, and amphibian cranial lines, form by elongation of a placode‐derived primordium that extends along the body *via* cell proliferation. The continuous sensory epithelial ridge then fragments, with each fragment differentiating into an individual neuromast.

It is already evident that head and trunk lines differ in their manner of development between different taxa. In zebrafish, both the head and trunk lateral lines develop as a migrating primordium deposits a series of neuromasts (Fig. 4A) (Iwasaki *et al*., 2020; Haas & Gilmour, 2006). By contrast, in a foundational study, Johnson (1917) showed that in a chondrichthyan (*Squalus acanthias*), both the head and trunk primordia elongate and subsequently fragment (Fig. 4B). This pattern of elongation and fragmentation is also found in the anterior lateral lines of a wide variety of non‐teleost osteichthyans (Allis, 1889; Beckwith, 1907; Harrison, 1903; Landacre & Conger, 1913; Winklbauer & Hausen, 1983; Modrell *et al*., 2011; Northcutt, Catania & Criley, 1994). However, the trunk systems in these osteichthyans exhibit similar migration and deposition patterns to zebrafish. For these reasons, we argue that the general condition for teleosts is unclear, and further note that all relevant data on anterior lateral line development are limited to studies of a single teleost sub‐group: the ostariophysans (Lekander, 1949; Landacre, 1910; Iwasaki *et al*., 2020).

**Fig. 5:**
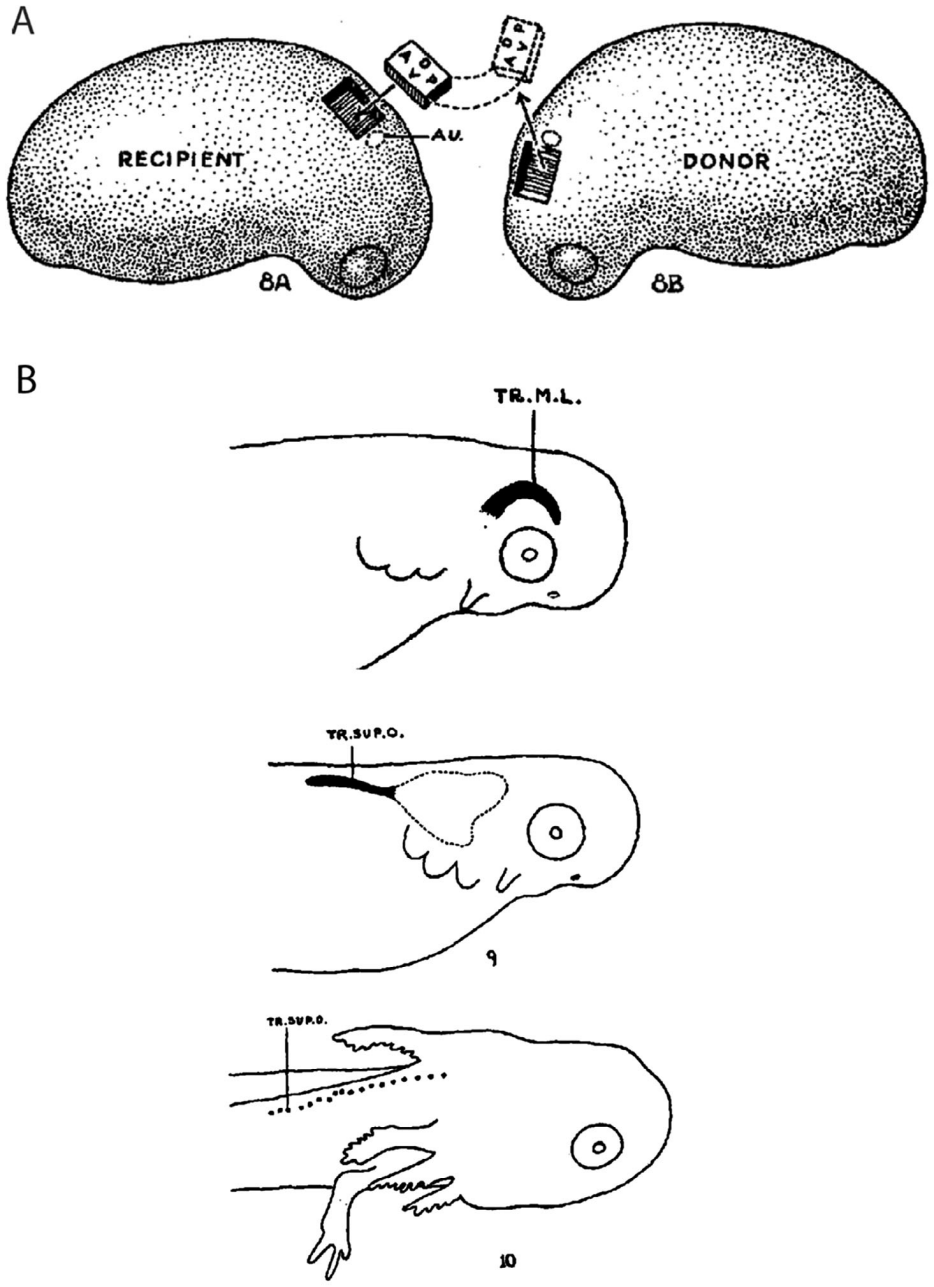
Stone's transplantation experiments. (A) Reproduction of fig. 8 of Stone (1928) schematizing a reciprocal transplantation approach, moving placodal ectoderm from preotic to postotic domains and *vice versa* between embryos of spotted salamanders (*Ambystoma punctatum*). (B) Reproduction of figs 2 and 9 of Stone (1928) *camera lucida* drawings showing that the preotic ectoderm migrated posteriorly leaving behind rosettes of hair cells when transplanted to a postotic domain. Conversely, postotic ectoderm elongated and formed ridges around the eye when transplanted to a preotic domain. Stone (1928) concluded that placodal ectoderm migrates or elongates based on its local environment.

The molecular mechanisms that underlie the development of lateral lines from primordia also remain unclear, with current knowledge derived exclusively from studies in zebrafish (Chitnis *et al*., 2012; Dambly‐Chaudière *et al*., 2007; Haas & Gilmour, 2006; Piotrowski & Baker, 2014, Neelathi, Dalle Nogare & Chitnis, 2018). These studies have demonstrated that the zebrafish posterior lateral line develops from a migrating primordium (Fig. 4A). The primordium follows a track of the chemokine Cxcl12 (C‐X‐C motif chemokine ligand 12) (Haas & Gilmour, 2006), which is laid down in a narrow stripe along the length of the underlying trunk paraxial mesoderm. Of note, the embryological experiments of Smith *et al*. (1990) suggest that a similar track may exist in axolotls, but in the overlying epidermis. Periodic deposition of neuromasts from the migrating primordium is dependent on a feedback loop between distinct Wnt (wingless related integration site) and Fgf (fibroblast growth factor) domains within the primordium (Aman, Nguyen & Piotrowski, 2011). However, there are no data yet on whether trunk lateral lines in other taxa that are deposited by migrating primordia (e.g. in amphibians, see Smith *et al*., 1990) follow similar chemokine tracks, nor whether Wnt/Fgf signalling are involved. Elongating primordia (Fig. 4B) might use entirely different molecular mechanisms to achieve neuromast distribution, including specific spatiotemporal patterns of gene expression. Surprisingly, however, the classic embryological experiments of Stone (1928) revealed that the head and trunk lateral line primordia of *Ambystoma punctatum* (axolotl) are functionally interchangeable (Fig. 5). However, it is unclear from figs 9 and 10 in Stone (1928) whether the anterior primordium, when transplanted to the trunk, migrated like a normal trunk primordium or instead elongated and fragmented. In either case, Stone's (1928) findings suggest that extrinsic signals from the surrounding cranial or trunk tissues must play a major role in patterning the placodal primordia and the lines that emerge from them.

## IV. NORTHCUTT'S PARADIGM

**Fig. 6:**
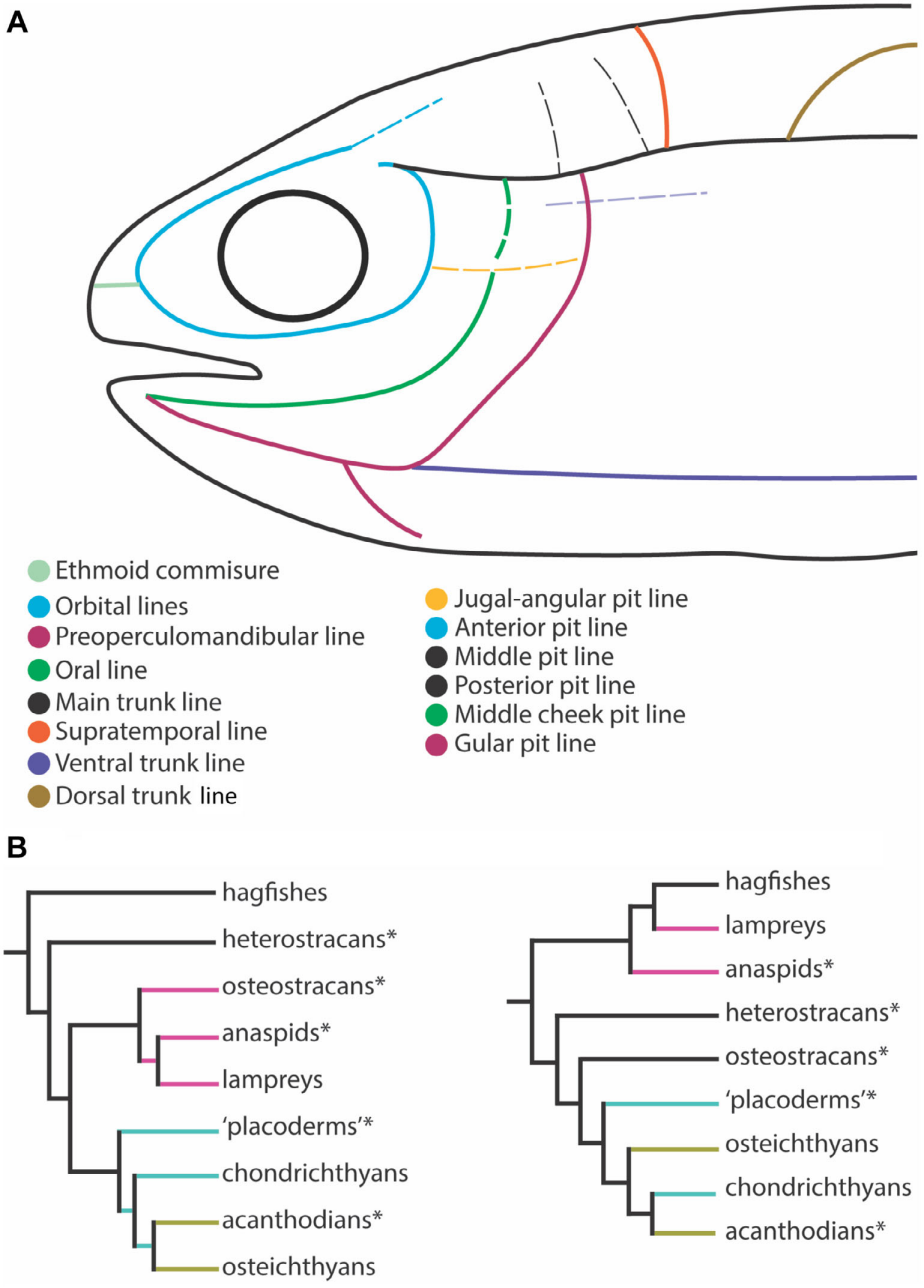
Ancestral gnathostome lines from Northcutt (1989) and transformed hypotheses of vertebrate interrelationships. (A) Northcutt's proposed morphotype for ancestral gnathostome head and trunk lateral lines [after Northcutt (1989), fig. 3.3D]. (B) Cladogram of fossil and living vertebrate relationships after fig. 3.1 in Northcutt (1989). (C) Taxa from B re‐distributed according to current phylogenetic consensus (Miyashita *et al*., 2019). Terminal branches are colour coded to aid comparison.

Northcutt (1989) provided the first cladistic and rigorously comparative analysis of lateral line evolution. His study combined embryological data from *Ambystoma mexicanum* with palaeontological and anatomical data from diverse sources (Northcutt, 1989; Northcutt *et al*., 1994) with the aim of reconstructing the ancestral gnathostome lateral line condition. Northcutt (1989) hypothesized an ancestral gnathostome with a set of orbital, mandibular and cheek lines (Fig. 6A). He further argued that these lines arise from six placodes and are innervated by a corresponding set of six placodal nerves. Based on this posited ancestral condition, Northcutt (1989) hypothesized that all diversity evident in extant vertebrate lateral lines resulted from descent with differential loss.

In this respect, Northcutt's work developed ideas put forward in earlier studies (Holmgren, 1942; Holmgren & Pehrson, 1949). However, Holmgren & Pehrson (1949) based their evolutionary scenario on a †heterostracan ancestor extrapolated from a theoretical vertebrate archetype. At that time, †heterostracans provided the earliest available information on fossil vertebrate conditions, hence their significance in these mid‐20th century studies. As noted in Section II (Fig. 2H), †heterostracan head shields bear a network of longitudinal and transverse lines. Therefore, they theorized that with the advent of a jaw, components of this network extended onto the new anatomical territory of the mandible. Holmgren (1942) and Holmgren & Pehrson (1949) connected their ancestral condition with “advanced types”, representing conditions in extant forms *via* a series of hypothetical intermediates. Unlike Northcutt's (1989) hypothesis, they included no explicit arguments for gains or losses at nodes in the vertebrate phylogeny. Moreover, they were influenced by the idea that extant “lower vertebrates”, such as the frilled shark *Chlamydoselachus anguineus* and lungfishes, more closely approached these primitive or archetypical states of vertebrate anatomy (Allis, 1923).

By contrast, Northcutt (1989) aimed to reconstruct trait history, generate hypotheses of evolutionary developmental mechanisms, explore form–function relationships, and identify knowledge gaps, all within an explicit tree‐based framework (Fig. 6B). It is important to note that at this time Northcutt had access to a limited set of fossil data (Moy‐Thomas & Miles, 1971). However, since 1989, our best estimates of early vertebrate interrelationships and evolution have changed drastically (Fig. 6C). Over 30 years' worth of discoveries have filled gaps in the fossil record in unexpected ways, while generating new questions. New phylogenies of early vertebrates have changed our fundamental understanding of character evolution. The notion of “higher” and “lower” taxa has been jettisoned, and *C. anguineus* is neither a living fossil nor a living ancestor. Similarly, it is also clear that cyclostomes (lampreys and hagfish) are far from primitive. Nevertheless, several of the knowledge gaps identified by Northcutt (1989) remain unfilled such as information on the comparative innervation and development of cyclostome lateral lines.

Importantly, Northcutt's (1989) analysis provided an explicit, testable hypothesis: that lateral lines evolved *via* a pattern of gradual reduction, and that traces of this ancestral condition are manifest in the distribution of canals, pit lines, and superficial neuromasts of extant taxa. In the next section, we explore this hypothesis in the light of updated phylogenies (Fig. 6C), acknowledging both stable and unstable areas of the evolutionary tree.

## V. NEW TREE, NEW SYNTHESIS

### (1) Vertebrate phylogeny revised

**Fig. 7:**
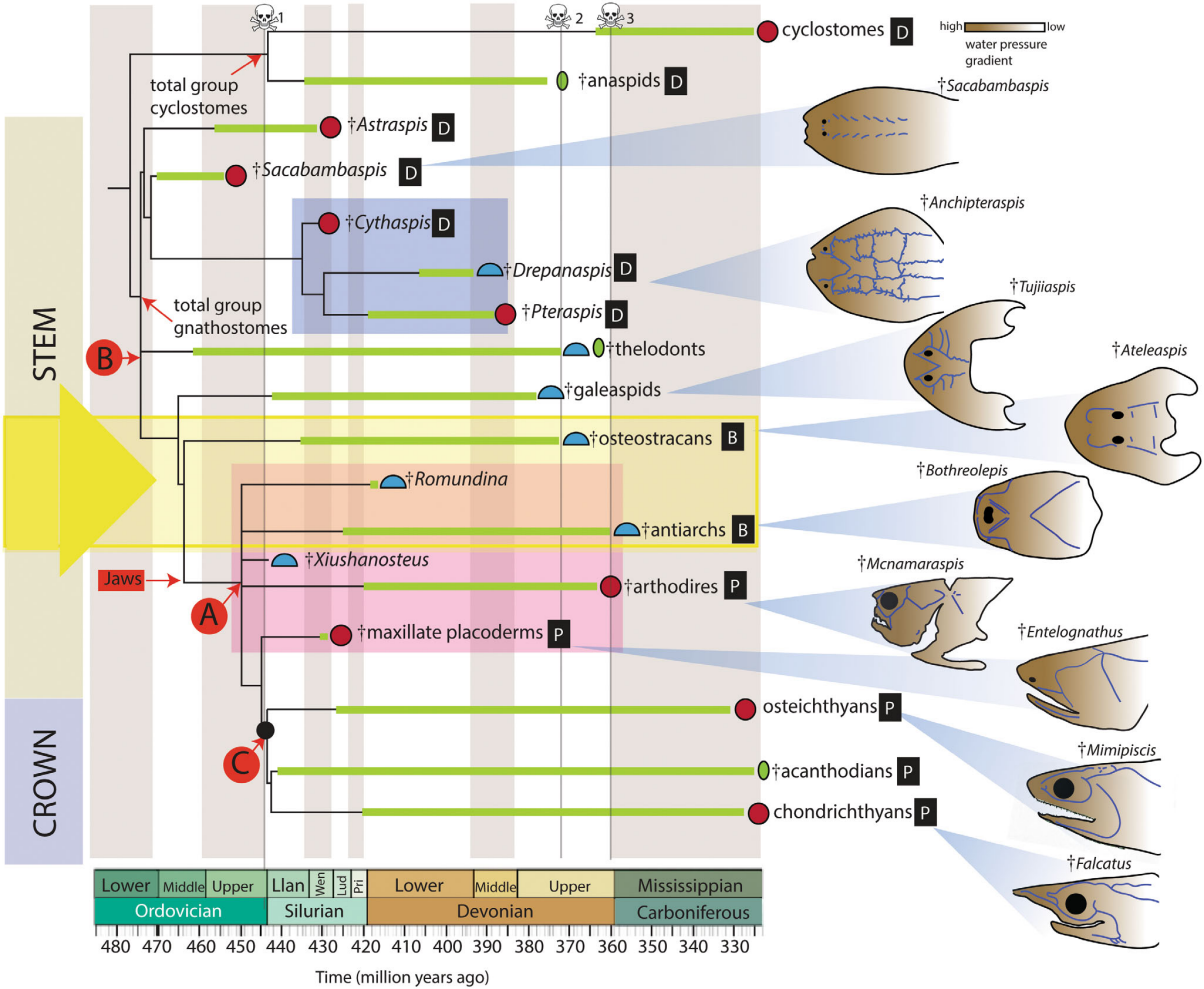
Early vertebrates and lateral line evolution. Time‐calibrated phylogenetic synthesis after King *et al*. (2017), Miyashita *et al*. (2019) and Zhu *et al*. (2022). Crania sketched with lateral lines (blue) after Gagnier *et al*. (1986), Elliott & Mark‐Kurik (2005), Gai *et al*. (2020), Stensiö (1932), Graham‐Smith & Parrington (1978), Choo (2012), Long (1995) and Lund (1985). Colour shading on crania represents hypothetical water pressure gradients. Graded triangles connect crania to position on tree. Yellow arrowed box is zone of major remodelling of lateral lines. Thick green lines represent taxon ranges. Magenta box captures “placoderms”; blue box captures heterostracans. Body shape indicated by symbols at branch tips: crimson circles are body shapes approximately circular in cross section; blue half circles are dorsoventrally flattened body shapes; green ovals are laterally flattened body shapes. Black boxed letters indicate inferred life habit (B, benthic; D, demersal; P, pelagic). Daggers represent extinct clades. Skull and crossbones denote extinction events: 1, End‐Ordovician; 2, Kellwasser; 3, Hangenberg (End‐Devonian). Black circle denotes crown node. Polytomies A and B (in red circles) represent unresolved branching within “placoderms” and jawless stem gnathostomes, respectively. Advent of jaws (red box) as indicated. Simple calibration aligning taxon ranges to stratigraphic timescale is based on the International Commission on Stratigraphy, v 2023/09.

Figure 7 presents a summary of the most recent phylogenetic trees of early vertebrates. It is important to recognize that this is not a formal supertree (Gordon, 1986; Bininda‐Emonds, Gittleman & Steel, 2002). Challenges to the consensus that underpinned Northcutt's phylogeny (Fig. 6B; fig. 3.1 in Northcutt, 1989) first emerged in Forey & Janvier (1993) and were further developed in Janvier's *Early Vertebrates* (see his “odd trees” in fig. 9.1 in Janvier, 1996). Changes cover all clades involved in this topology. To set the stage for our discussion of lateral line macroevolutionary patterns in Section V.3, we describe some important changes to the topology and identify some of the unresolved relationships.

In contrast to Northcutt's (1989) hypothesis (compare Figs 6B and 7) †“acanthodians” are now recognized as early chondrichthyans (Brazeau, 2009; Davis, Finarelli & Coates, 2012; Coates *et al*., 2018). This discovery of a stem group has vastly enriched the entire chondrichthyan lineage but depopulated the osteichthyan stem. The few remaining stem osteichthyans are contentious in the sense that they have been equivocally interpreted as stem or crown group members, with either actinopterygian or sarcopterygian affinities (Basden *et al*., 2000; Lu *et al*., 2016).

†“Placoderms” (magenta box in Fig. 7) are the earliest jawed vertebrates in the fossil record. Taxonomically diverse and morphologically disparate, they might not constitute a natural group. If instead †“placoderms” constitute a grade, meaning clades branching successively from the stem, then they can provide a series of snapshots of trait evolution leading to modern jawed vertebrates (Dupret *et al*., 2017; Zhu *et al*., 2016, 2013; Zhu *et al*., 2022). Alternatively, if a large monophyletic rump group of †“placoderms” persists, then they represent an independent evolutionary radiation of early jawed fishes. In this case, they must have converged on several morphologies shared with modern clades. Both hypotheses are current – monophyly (natural group, see King *et al*., 2017) and paraphyly (Brazeau & Friedman, 2015; Brazeau *et al*., 2023); for this reason, we include polytomy A in Fig. 7.

†*Entelognathus* is the first maxillate †“placoderm”, meaning it has a maxilla, like osteichthyans. But perhaps more significantly, it is also the first †“placoderm” found to have lateral lines on the jaws, thus bearing out Northcutt's predictions (Northcutt, 1989, p. 69). Among the †“placoderms”, †*Entelognathus* and other maxillate forms are now hypothesized to be sister groups to the extant gnathostome evolutionary radiation.

In contrast to the instability of †“placoderm” relationships, cyclostome monophyly is now resolved on the basis of both molecular and morphological evidence (Furlong & Holland, 2002; Kuraku *et al*., 1999; Heimberg *et al*., 2010; Miyashita *et al*., 2019). As a consequence of this monophyly, early jawless fishes have been redistributed across cyclostome and gnathostome stems. The groups are shown in Fig. 7, although uncertainty persists (see polytomy B). *Contra* Northcutt (1989), †osteostracans (Stensiö, 1932; Janvier, 1985; Sansom, 2009) are now regarded as the sister group to jawed vertebrates (Forey, 1995; Donoghue & Smith, 2001; but see also Gai *et al*., 2011). †Heterostracans and their likely relatives (Fig. 7; blue box) provide additional and abundant data on what appears to be an independent evolutionary radiation of stem‐gnathostomes (Randle, Keating & Sansom, 2022). †Thelodonts (Ferrón & Botella, 2017; Wilson & Märss, 2009; Wilson & Caldwell, 1993, 1998) remain the least fixed group in early gnathostome phylogeny, constituting a “wild card” taxon (Nixon & Wheeler, 1992) because of their incomplete preservation, often consisting of little more than assemblages of scales.

The chronology of branching points is based on a series of minimum dates estimated from the fossil record, as determined by taxon ranges (Fig. 7; thick green lines). Recent discoveries (Zhu *et al*., 2022) have confirmed phylogenetic predictions (Brazeau & Friedman, 2015; Andreev *et al*., 2016; Coates *et al*., 2018) that crown gnathostomes date from at least the early Silurian (440 million years ago). This has consequences. Figure 7 shows that a minimum hypothesis of the age of the last common ancestor of extant gnathostomes is pushed back in time, earlier than the earliest occurrences of the vast majority of †“placoderms” and most of their jawless outgroups. The key question now concerns the implied missing record of early vertebrates: how many groups of jawed and jawless fishes originated before or after the extinction event at the Ordovician–Silurian boundary (Harper, Hammarlund & Rasmussen, 2014; Servais & Harper, 2018)? Futhermore, how might these unknown groups transform our current best hypotheses of morphological evolution, including lateral lines?

### (2) New hypotheses of lateral line evolution

The most arresting feature of cranial lateral line evolution depicted in Fig. 7 is the breakdown and reassembly of the lateral line networks near the origin of gnathostomes (Fig. 7, yellow box). An overview of lateral line conditions in groups such as †heterostracans and †galeaspids reveals an abundance of reticulating lines with numerous twigs and branches ramifying across the dorsal surface in a distinctively postorbital position. The preorbital domain in most of these fishes is short and devoid of branching lateral lines. In jawed vertebrates, the situation is quite different: the body of the lateral line network has switched from a postorbital to an orbital/preorbital position. Lines circumnavigate the orbits, traverse the snout, and populate the cheek and jaws. Crucially, this evolutionary episode of reassembly, remaking and remodelling, starts before the origin of jaws.

Our evolutionary scenario shows early and divergent trajectories of sensory system elaboration. This contrasts with traditional narratives in which systems and characteristics evolve, usually towards humans, in a linear fashion. In Northcutt's (1989) framework, the path is one of descent with loss and modification. Most recently, Edens & Bronner (2025) reviewed the evolution of placodes and neural crest to reconstruct the serial assembly of peripheral sensory structures, but this too, is essentially a linear narrative.

Returning to Fig. 7, in our scenario, the earliest full elaboration of the anterior sensory line system is seen in †heterostracans and their relatives. This elaboration is also seen in the broad head shields of †galeaspids, but it is strikingly absent in the similarly shaped head shields of †osteostracans. Thus, †galeaspids and †osteostracans bracket the first signal of this transformation. In both, the general condition of the head shields is dorsoventrally flattened and broadly semi‐lunate with dorsally positioned orbits. However, the lateral line networks and other sensory systems are strikingly different. †Galeaspids show the elaborate, ramifying, postorbital patterns seen in †heterostracans and other allied groups, but †osteostracans have only a rudimentary array of superficial lines. And this signature persists in the earliest jawed fishes: the †“placoderms”. Here, the general pattern of lines radiates out from the centre of the postpineal domain, much like †galeaspids, but lateral line occupation of the cheek region and lower jaw regions is limited. Both of these territories are evolutionary novelties relative to jawless conditions. In this respect, †*Entelognathus* is unique among †“placoderms” in having lines on the lower jaw (Zhu *et al*., 2016 *contra* Stensiö, 1947). These data suggest a “phylogenetic lag”: new domains are not immediately occupied by the lateral line system.

Switching focus to extant clades, our new synthesis signals that, *contra* Northcutt (1989), the lateral lines of extant gnathostomes are not the end products of serial reductions from a maximally lined common ancestor (Fig. 6A). Instead, we propose that lateral line systems diversified independently in quite distinct trajectories among the jawless and jawed lineages. This raises the question of how this new scenario changes our estimates of general *versus* specialized conditions among the lateral lines of extant groups.

Among modern fishes and their extinct relatives, the infraorbital line emerges as the most stable component of the system. In living sharks and bony fishes, the infraorbital line consistently joins the supraorbital line behind the orbit (Figs 2C, D, O, P and 7). But the introduction of fossils like †*Mimipiscis* and †*Falcatus* (Fig. 7) shows that this connection is a convergent phenomenon (as noted in Section II.2). By contrast, supraorbital lines are more evolutionarily labile. Most †“placoderms” such as †arthrodires (Fig. 7) possess supraorbital and infraorbital lines indicating that these are likely a general feature for all jawed gnathostomes. Therefore, the absence of supraorbital lines in maxillate †“placoderms” (Zhu *et al*., 2013) such as †*Entelognathus* is probably derived.

The evolutionary hypothesis laid out in Fig. 7 also raises questions about lateral line placode evolution. Mandibular lines are considered a general feature of the cranial lateral line network. However, their first appearance in the lower jaws of taxa such as the maxillate †“placoderm” †*Entelognathus*, in the absence of any cheek canal, is likely important. In modern jawed vertebrates, the mandibular line is continuous with a preopercular line on the cheek (Fig. 1). Developmental data (Northcutt *et al*., 1994; Iwasaki *et al*., 2020) indicate that the mandibular and preopercular lines develop from a single anteroventral placode. This suggests a transformational hypothesis. First, the evolution of the mandible was followed by the *de‐novo* condensation of a new placode. Next, the developmental derivative of this placode invaded the new territory of the cheek. Currently, there are no data on whether any homologue of an anteroventral placode exists in extant jawless cyclostomes (hagfishes and lampreys).

The macromeric skulls of osteichthyans, that is skulls comprising large plates, have been presented as the result of correlated evolution between lateral lines and the dermal skeleton (Westoll, 1941; Pehrson, 1922). These skulls were previously thought to have evolved from a micromeric skull composed of tiny scales (Romer, 1966; Schultze, 1993). However, this general scenario is no longer supported by current hypotheses and data. First, micromeric skulls are now recognized as a derived feature, and are not ancestral to macromeric skulls (Friedman & Brazeau, 2013). Second, major features of the lateral line network persist through this shift from macromery to micromery. Third, in macromeric skulls such as our own, the general pattern of skull bones persists despite the complete and long‐standing absence of a lateral line system. For these reasons we argue that the respective pattern stabilities of cranial bones and lateral lines are independent to a greater degree than previously appreciated.

### (3) Functional implications and macroevolutionary pattern

Figure 7 shows how extinction events (skull and crossbones) map through time relative to the taxon ranges and branching events in this summary of vertebrate phylogeny. The origin of all major clades – both jawed and jawless – dates back to at least the early Silurian, and the early radiation of these lineages might have occurred in response to the end‐Ordovician extinction (Harper *et al*., 2014). Similarly, the foundations of extant vertebrate diversity, composed mostly of osteichthyans (tetrapods and actinopterygians) and chondrichthyans were laid in the aftermath of a pair of late Devonian extinctions, the Kellwasser and Hangenberg events (Sallan & Coates, 2010). Notably, both tetrapods and actinopterygians diversify into new body shapes, new abundance, and new habitats. The so‐called “Age of Fishes”, formerly the Devonian “pre‐tetrapod” vertebrate world now ranges from the end‐Ordovician to the end‐Devonian, a span of some 80 million years. Moreover, throughout most of this period jawless and jawed fishes overlapped. There is no rapid elimination of jawless taxa, but quite how this ichthyological Eden was maintained remains to be determined.

The acquisition of crown gnathostome characters, including a heterocercal tail, muscular pectoral and pelvic fins, a third semicircular canal of the inner ear, a jaw, and dentition, enabled increasingly active swimming and a wider range of feeding strategies. These changes are associated with the most successful of what appear to have been several ecological excursions into the water column by different early lineages, and likely underpinned the vertebrate component of the Devonian Nekton Revolution (DNR) (Klug *et al*., 2010; for alternative views of timespan and significance of the DNR see Whalen & Briggs, 2018). Crucially, all of these transformations accompany the major reorganization of the cranial lateral line network.

The DNR (Klug *et al*., 2010), encapsulating increased occupation of the water column by nektonic and demersal clades (invertebrate and vertebrate), fundamentally changed marine ecosystems and food webs. Aside from changes already outlined for vertebrates in general, this shift is also reflected in body cross‐sectional outlines. Figure 7 shows transitions from broad, dorsoventrally flattened conditions (blue half‐circles) to more laterally compressed profiles (crimson circles). Among the jawless groups, both †anaspids and †thelodonts have bodies with round to laterally compressed cross sections (green ovals) suggesting life in the water column, and swimming modes in these taxa have been modelled as such (Ferrón & Botella, 2017; Ferrón & Donoghue, 2022; Ferrón *et al*., 2020). However, the extent to which these fishes could sustain occupancy of the water column, as well as their ability to evade predators, is unknown.

For other early jawless gnathostomes, such as †*Astraspis*, body shapes indicate that they experienced downward thrust, consistent with demersal (Fig. 7, taxa marked D) or strictly benthic (Fig. 7, taxa marked B) habits. All of these fishes likely fell victim to invertebrate predators that had already begun occupying the water column (e.g. †eurypterids and †radiodonts; Klug *et al*., 2010). For these reasons, we suggest that the extensive dorsal postorbital lines were adaptations for predator evasion and escape, analogous to arthropod looming receptors (Rind *et al*., 2016). The general absence of lateral lines at the front of the head implies that these organisms were not gaining extensive sensory input from bulk water flow (Mogdans, 2019). Exceptions to this rule include heterostracans with extended rostra which very likely served as sensory arrays (e.g. *Errivaspis*; White, 1935). We acknowledge that many of these early jawless fishes have very little preorbital territory, but what is absent is a concentration of sensory lines and pits surrounding the orbitonasal and oral complex (e.g. *Sacabambaspis*). The lines in these cases are distributed evenly across the length of the headshield. The broadly limited sensitivity of these fishes to current strength and direction may have restricted swimming efficiency and sophistication (i.e. they were unlikely to have schooled or exhibited other coordinated group behaviours) and, by extension, limited their habitat occupation. By contrast, the evolution of complex mechanosensory systems located rostrally, combined with muscular paired fins, provided the capability to become fully pelagic (Fig. 7, taxa marked P), perform enhanced swimming manoeuvres, exhibit novel behavioural responses, and establish new ecological niches.

This sensory elaboration probably happened in a stepwise manner. †Osteostracans (Figs 2F and 7) show that the evolution of at least one pair of muscular fins, a heterocercal tail, and large‐scale remodelling of sensory systems preceded the advent of jaws. †Osteostracans and †galeaspids have similar body shapes (Janvier, 1996), but †osteostracans have exceptionally reduced lateral lines (Stensiö, 1932; Janvier, 1974). However, their peculiarly elaborated vestibular system is connected to large dorsal and rostral sensory fields. We suggest that these functioned similarly to the rostral lines of crown gnathostomes to perceive bulk water flow towards the front of the head (Janvier, 1985). A similarly rudimentary lateral line system, but lacking any trace of sensory fields, is seen in †“placoderms” thought to represent primitive conditions (†antiarchs and †romundinids) (Denison, 1978). The modest jaws of these early branching clades precluded any possibility of macropredation, suggesting that jaws and teeth were only later co‐opted for predatory behaviour. Notably, even the canal‐bearing jaws of the maxillate †“placoderm”‐grade antecedents of crown‐group gnathostomes are toothless (Zhu *et al*., 2013). In summary, lateral lines are part of a major reorganization of sensory systems that both preceded and overlapped a vertebrate body plan that we now recognize as fundamental to modern gnathostomes.

## VI. FUTURE DIRECTIONS: AVENUES FOR INTEGRATIVE RESEARCH

An integrative approach, combining palaeontological, anatomical and developmental data is essential to fill the outstanding gaps in our understanding of lateral lines. Here we lay out an agenda of five themes deserving future work.

A key question is the relationship between lateral lines in jawless and jawed vertebrates: how do the lines in living agnathans relate to those in gnathostomes? Resolving this involves study of the only extant jawless vertebrates with extensive lateral lines: lampreys. While palaeontologists have attempted to address this question by comparing osteostracans and lampreys (Janvier, 1974), current phylogenetic consensus places these two lineages distant from each other. In addition, embryological evidence regarding the development of lateral lines in lampreys is lacking and would help reveal the placodal origins of lamprey lateral lines. This in turn would clarify the homologies between jawless and jawed vertebrate lateral lines and reveal whether lampreys have a precursor of the vertebrate anteroventral placode.

Another key area of research is the origin and development of lateral lines in fishes with extreme morphologies (see Section II.4). For example, it is unclear which placode produces the novel pleural lines on batoid fins, nor do we know how they grow posteriorly. Molecular assays including antibody staining for proteins on large embryos (Masselink & Tanaka, 2023), and hybridization chain reactions (HCRs) to assay gene expression (Dirks & Pierce, 2004) would help trace the development of these lines at high resolution in fixed specimens. Combined with a vital dye labelling approach [e.g. using DiI (1,1′‐dioctadecyl‐3,3,3′,3′‐tetramethylindocarbocyanine); Gillis *et al*., 2012], this would illuminate the specific origin and homologies of these novel lines.

The relationship between the lateral line and the surrounding dermal skeleton (Section III) presents a further area ripe for investigation. While advances in tomographic imaging have vastly improved our knowledge of fossil dermal skeletons (Giles *et al*., 2013; Keating & Donoghue, 2016), none of these studies has focused on lateral lines. Moreover, experimental embryological evidence is needed to follow up on preliminary work looking at the relationship between the lateral line and other tissue types (Smith *et al*., 1990).

There are crucial gaps to fill within the fossil record, especially the missing Silurian ranges of jawless and jawed vertebrates (Section V.1). Resolution of †“placoderm” phylogeny, with further clarity on whether the clade forms a monophyletic group, will inform patterns of trait evolution in crown jawed gnathostomes. In particular, this would add detail to the evolutionary phase in which the lateral line system was remodelled to the condition at the base of extant jawed vertebrates (Fig. 7; yellow box).

The establishment of transgenes labelling zebrafish lateral lines (Haas & Gilmour, 2006) has allowed unprecedented access to spatiotemporal patterns of development in this system. Using genetic, pharmacological or embryological approaches to ablate different tissue types in model systems like zebrafish or *Xenopus* would help explore the interaction between placodal lines and adjacent tissues like neural crest or underlying mesoderm. We lack a mechanistic understanding of how migrating *versus* elongating primordia are patterned. Antibody staining, combined with other morphological visualization techniques including scanning electron microscopy and paraffin histology would help advance our understanding of this difference.

Finally, vital dyes like TMRE (tetramethylrhodamine, ethyl ester, perchlorate) or DASPEI (2‐(4‐(dimethylamino)styryl)‐N‐ethylpyridinium iodide) enable the visualization of maturing hair cells and thus neuromasts through the entirety of ontogeny in an unprecedented variety of vertebrates (Pisano *et al*., 2014; Esterberg *et al*., 2013).

## VII. CONCLUSIONS

(1) Lateral line systems were drastically remodelled during early vertebrate evolution. The lateral lines of the earliest jawless fishes were elaborated predominantly in their postorbital and dorsal domains. However, the lateral lines of early and modern jawed vertebrates are elaborated orbitally and preorbitally. This sequence of change implies an episode of reduction followed by re‐modelling and rebuilding. This reorganization correlates with, but slightly precedes, the origin of jaws. This phylogenetic pattern is not recapitulated in ontogeny; neither is it congruent with previous hypotheses of descent *via* simplification from an elaborate ancestral pattern. Such major change in lateral line distribution implies a functional shift, which is likely related to changes in behaviour and ecology.
(2) Lateral lines of the cranial and trunk domains develop differently in different taxa. Moreover, cranial and trunk lateral lines in the same taxa may result from alternative developmental processes. Two primary modes of lateral line development are the deposition of neuromasts by a migrating primordium and the fragmentation of an elongating ridge. These contrasting modes are manifest across head/trunk boundaries but also across taxonomic divides. Osteichthyan and chondrichthyan patterns of line development differ, but have yet to be documented in detail and the polarity of evolutionary change has yet to be determined.
(3) Classic experiments suggest that neuromasts do not have an inductive effect on the ossification of dermal bones. But canal bone, an important component of osteichthyan skeletal histology, has been overlooked. Recent ablation experiments in zebrafish have provided some evidence of a role for neuromasts in canal bone induction. For these reasons we argue that the relation of canals to dermal bone growth and pattern in general is an area ripe for examination.
(4) There is an apparent hierarchy of cranial sensory system development. Changes in lateral line distribution appear to be secondary to changes in the location of orbits and nostrils, such that the lines adjacent to these features are redeployed to accommodate changes in visual and olfactory systems. This hierarchical relationship has a phylogenetic component and seems to be most strongly expressed in osteichthyans. It is not yet established whether this relationship is the result of differences in developmental mechanism *versus* developmental timing.
